# Retinoic Acid Metabolism-Related Enzyme Signature Identified Prognostic and Immune Characteristics in Sarcoma

**DOI:** 10.3389/fcell.2021.780951

**Published:** 2022-02-03

**Authors:** HuaiYuan Xu, JinXin Hu, YiJiang Song, HongMin Chen, YanYang Xu, ChuangZhong Deng, Hao Wu, GuoHui Song, JinChang Lu, QinLian Tang, LiangPing Xia, Jin Wang, XiaoJun Zhu

**Affiliations:** ^1^ Department of Musculoskeletal Oncology, Sun Yat-Sen University Cancer Center, Guangzhou, China; ^2^ State Key Laboratory of Oncology in South China, Sun Yat-Sen University Cancer Center, Guangzhou, China; ^3^ VIP Department, Sun Yat-sen University Cancer Center, Guangzhou, China

**Keywords:** retinoic acid metabolism, sarcoma, LASSO-penalized cox regression, immune, CD8^+^ T cell

## Abstract

Growing evidence indicates a link between retinoic acid (RA) metabolism and sarcoma progression or immunity in laboratory studies. However, a comprehensive analysis of RA abnormality in the sarcoma population is still lacking. Herein, we systematically analyzed the molecular features of 19 retinoic acid metabolism-related enzymes and sarcoma patients’ clinical information based on TCGA/TARGET/GSE datasets. We identified two RA expression subtypes, which were related to distinct clinical survival outcomes and exhibited different biological features. Gene set enrichment analysis indicated a set of immune pathways were enriched in G1 while oncogenic pathways were enriched in G2. Immune cell infiltration analysis using the TIMER algorithm revealed more CD4^+^ and CD8^+^ T cell infiltration in G1 subgroups than in G2. Moreover, we generated a seven genes signature to predict the RA metabolism index based on the LASSO-penalized Cox regression model. Survival analysis demonstrated the significant prognostic differences between high- and low-risk groups among different bone and soft tissue datasets. A higher risk index was associated with less T cell CD8^+^ infiltration. The predictive ability of the RA risk score was validated in 71 bone or soft tissue sarcoma clinical samples. These results indicated that RA-based classification could distinguish sarcoma patients with different clinical outcomes and immune statuses, which may help to explore better treatment decision-making for sarcoma patients.

## Introduction

Sarcoma is a broad set of bone and soft tissue neoplasms, with the common characteristic of being derived from mesenchymal cells (Hatina et al., 2019). Clinical management of bone and soft tissue sarcoma consists mainly in surgical resection, with adjuvant therapies that depend on the Fédération Francaise des Centres de Lutte Contre le Cancer (FNCLCC) grading system, which was defined more than 20 years ago and is still the most commonly used (Trojani et al., 1984; Guillou et al., 1997). It is based on indirect measuring of oncogenic changes within the tumor, including tumor differentiation, necrosis, and mitotic index. However, tumor intrinsic qualities alone do not provide sufficient information for predicting the clinical course of the disease.

It is increasingly recognized that the tumor immune microenvironment (TIME) is an important prognostic and predictive indicator for bone and soft tissue sarcoma (Chen et al., 2020; Deng et al., 2020; Song et al., 2021). Results of clinical studies have also shown that a small proportion of sarcoma patients show a sustained response to immunotherapy (Tawbi et al., 2017; D'Angelo et al., 2018). Nevertheless, patients with even the same sarcoma subtype may have different tumor immune infiltrates, and showed different treatment responses, suggesting the traditional histological classification is limited in identifying patients with effective immunotherapy. Hence, determination and comprehensive understanding of the molecules that affect the immune microenvironment of sarcoma is needed and may help to improve risk stratification and better decision-making for sarcoma patients.

Recently, multiple lines of evidence pointed to retinoic acid (RA) and indicate that RA signaling is a critical regulator to remodeling the sarcoma immune microenvironment. In representative mouse models of the three main genetic categories of sarcoma, Devalaraja et al. found that sarcoma cell-derived RA inhibited the differentiation of tumor infiltrated monocytes into antigen-presenting dendritic cells, and instead promoted their development into immunosuppressive tumor-associated macrophages. Such immunosuppressive microenvironment inhibits the infiltration of antitumor CD8^+^ T-cells and is associated with poor response to treatment and poor prognosis of sarcoma (Song et al., 2020; Balkwill, 2020). Likewise, Yuning J. et al. traced clonal dynamics in the sarcoma mouse model then found that RA enzymes expression was dysregulated in the sarcoma primary tumors before the formation of metastases (Tang et al., 2019). As for immunotherapy, Adrienne H. found that RA plays an important role in improving CAR therapy efficacy in sarcoma xenograft models (Long et al., 2016). However, most of these studies are limited to cell or animal experiments, and there is a paucity of data based on the sarcoma population.

In this study, we systematically analyzed the molecular features of 19 RA metabolism-related enzymes and revealed two subtypes with distinct metabolic status and prognostic value. Transcriptome and genome traits of distinct RA-based subtypes were also interpreted, Gene set enrichment analysis indicated that several immune pathways were enriched in the subtype G1 subgroup, suggesting that higher infiltration of immune cells which was associated with prolonged survival. Whereas, the G2 subgroup was related to oncogenic pathways such as MYC targets and KRAS signaling. Additionally, we generated a seven genes signature to predict retinoic acid metabolism index based on the LASSO-penalized Cox regression model. Immune cells including CD8^+^ T cells, Treg cells, monocytes, and macrophages showed different abundance between these groups. Moreover, correlations between RA-based risk score and CD8^+^ cells were assessed and verified based on 71 sarcoma primary tumors. Together, this work revealed that the RA signaling modification played a non-negligible role in sarcoma. Evaluating the RA signaling pattern of sarcoma patients will contribute to the improved efficiency of personalized therapy.

## Results

### Expression Patterns of RA Metabolism Regulators in Sarcomas

**FIGURE 1:**
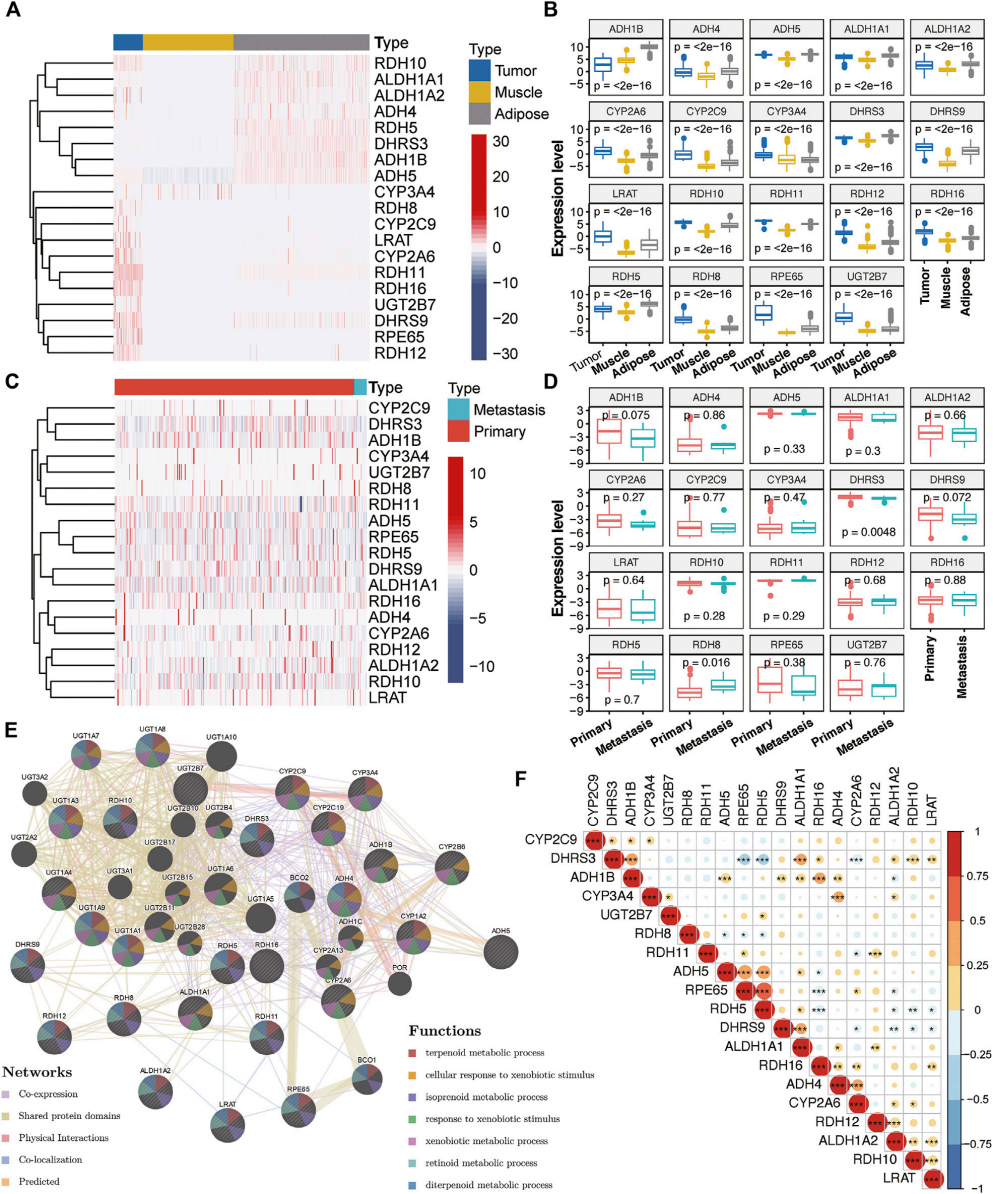
Expression of RA metabolism-related enzymes in SARC. Heatmap **(A)** and boxplot **(B)** of expression levels of the RA metabolism-related enzymes (normal sample vs. tumor sample). Heatmap **(C)** and boxplot **(D)** of expression levels of the RA metabolism-related enzymes (primary sample vs. recurrent sample) **(E)** PPI network and pathway analysis of the RA metabolism-related enzymes **(F)** Spearman correlation analysis of the RA metabolism-related enzymes.

With the mRNA expression data stored in TCGA and GTEx database, we examined the relationship between RA metabolism-related enzymes and the clinical features. The results for 19 enzymes expression and relevant clinical features were displayed in the heatmap and boxplot (Figure 1). Due to that there are only two normal samples for sarcomas in TCGA, we collected the muscle and adipose tissues in GTEx as normal controls. The sample size for tumor samples, adipose tissue samples, and muscle tissue samples were 263, 1,204, and 803, respectively. Among the 263 tumor samples, 13 samples were annotated as metastasis samples, while the others were primary tumor samples. Figures 1A,B indicate that the majority of RA metabolism-related enzymes were differentially expressed between sarcomas and normal tissues. Compared with normal tissues, the expression levels of CYP3A4, RDH8, CYP2C9, LRAT, CYP2A6, RDH11, RDH16, UGT2B7, DHRS9, RPE65, and RDH12 were elevated, while the expression level of RDH10, ALDH1A1, ALDH1A2, ADH4, RDH5, ADH1B, and ADH5 were decreased. However, few enzymes showed a significant correlation with recurrence (Figures 1C,D). Only DHRS3 was upregulated in primary tumors and RDH8 was upregulated in recurrent tumors. We then further dissected the regulation network and pathways of RA metabolism-related enzymes based on expression patterns, sublocations, and protein-protein interactions (Figure 1E). RA metabolism was found to be related to several metabolic pathways such as terpenoid, isoprenoid, retinoid, diterpenoid, glucuronate, and uronic acid metabolic process. Finally, we explored the correlation of RA metabolism-related enzymes (Figure 1F). Most enzymes were positively correlated, especially DHRS3, which exhibits the most positive correlation with other enzymes.

### Identification of RA Based Subtype in Sarcoma

**FIGURE 2:**
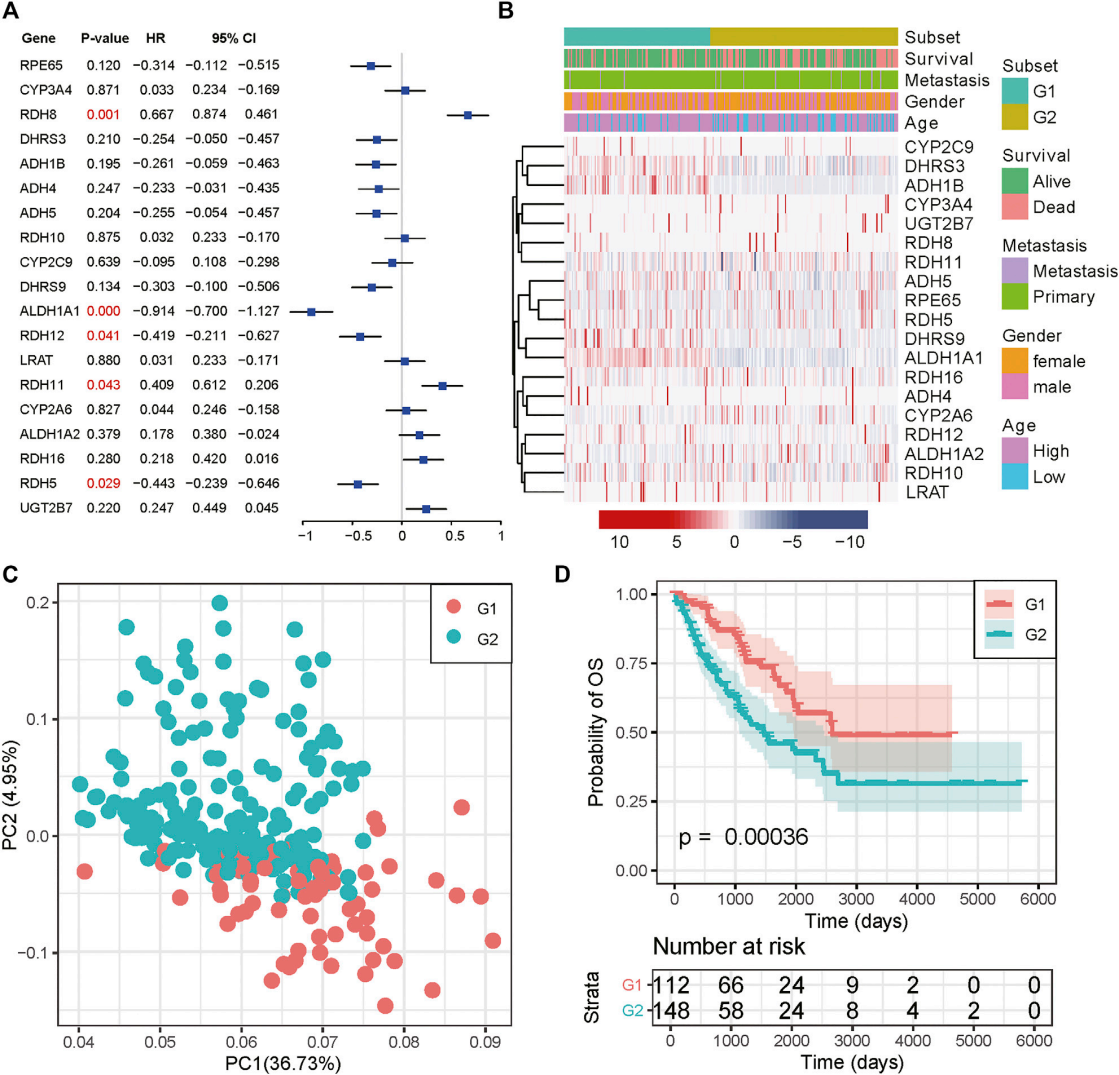
Consensus cluster of SARC samples. **(A)** Forest plot of the RA metabolism-related enzymes. **(B)** Heatmap and clinicopathologic features of the two clusters (G1/2) defined by the RA metabolism-related enzymes consensus expression. **(C)** Principal component analysis of the total mRNA expression profile in the TCGA dataset. **(D)** Kaplan–Meier OS curves for different subgroups.

We then examined the prognostic value for all RA metabolism-related enzymes. ALDH1A1, RDH5, and RDH12 were positively related to a better prognosis, while RDH8 and RDH11 indicated poor prognosis (Figure 2A). These data suggest that different RA-associated gene expression has different prognostic implications for sarcoma patients and that sarcomas based on different forms of RA-associated gene expression may have different biological behaviors as well as characteristics of the tumor immune microenvironment. The mechanisms that drive human osteogenic and soft tissue sarcoma tumorigenesis are in three main categories: 1) translocations generating fusion oncogenes, 2) mutations in tumor suppressors or oncogenes, and 3) genomic instability without a consistent mutation (Taylor et al., 2011). All three representative genetic abnormalities could be found in the TCGA-SARC dataset, such as synovial sarcoma (driven by SYT-SSX fusion oncogene), dedifferentiated liposarcoma (characterized by 12q13∼15 amplification), and undifferentiated sarcoma (lacking any defined line of differentiation) (Cancer Genome Atlas Research Network, 2017). Hence, to identify the RA expression profiles among sarcomas, we adopted an unsupervised hierarchical clustering method to generate the subtype of sarcomas based on the expression matrix of 19 enzymes in the TCGA-SARC dataset without any prior knowledge. The optimal number of clusters was determined using three parameters: The C index for the prognostic differences, the Silhouette index, and the Calinski–Harabasz criterion (Supplementary Table S1). As a result, 263 samples from the TCGA database were classified into two groups, which were then renamed G1 and G2 subgroups (Figure 2B and Supplementary Figure S1A). Principal component analysis (PCA) revealed a distinct expression pattern between these two groups (Figure 2C). Interestingly, the G2 subgroup mainly contained the sarcomas with elevated expression of RDH11, RDH8, RDH16, CYP2A6, and ALDH1A2, all of them were strongly or slightly correlated with poor survival. The genes highly expressed in the G1 subgroup were just the opposite, the majority of them were correlated with better survival. We then performed Kaplan-Meier survival analysis for these subtypes and noticed a significantly decreased overall survival (OS) status in the G2 subgroup compared to the G1 subgroup, suggesting that the 19 enzyme-based markers could indicate the prognostic risk level (Figure 2D). The median survival for G1 and G2 subgroups was 3.09 and 2.11 years. The detailed distribution of clinical variables for these patients was displayed in Supplementary Table S2. There is no significant difference in clinical variables between G1 and G2 subgroups.

### Transcriptome, Genome, and Immunity Traits of Distinct RA Based Sarcoma Subtypes

**FIGURE 3:**
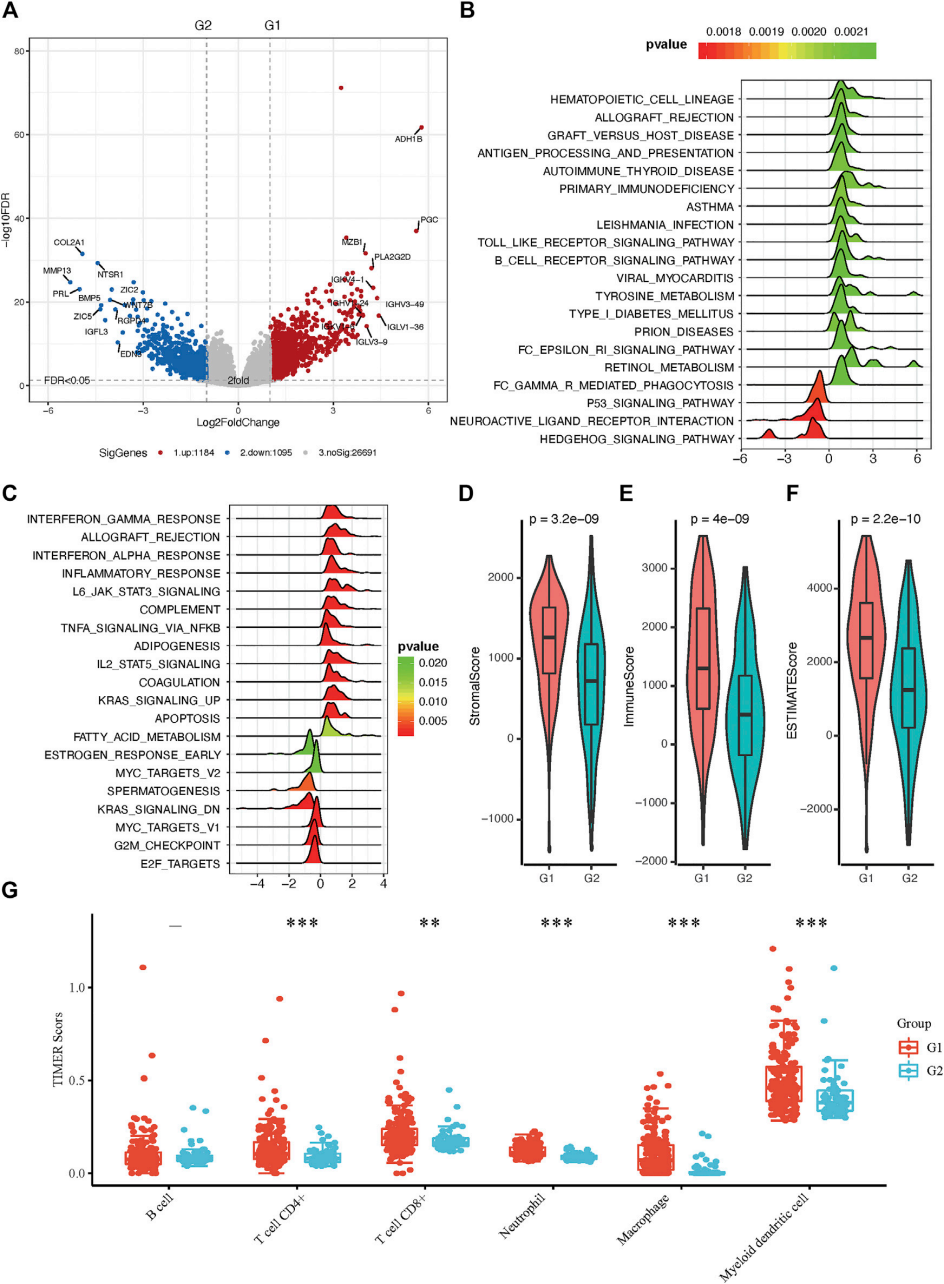
Molecular and immunity features of the two subgroups. **(A)** Volcano plot of identified DEGs. **(B)** Functional annotation of the DEGs with hallmark pathways. **(C)** Functional annotation of the DEGs with KEGG pathways **(D–F)** The immune microenvironment between the two groups. **(G)** Distribution of TIMER scores in sarcoma tissues, the horizontal axis represents different groups of samples, the vertical axis represents gene expression distribution, different colors represent different groups, upper left corner represents significance *p*-value test method. Asterisks represent significance levels (*p < 0.05, **p < 0.01, ***p < 0.001).

To better understand the molecular mechanisms between RA metabolism regulation and sarcomas. We performed differential expression analysis and identified 2,279 differentially expressed genes (DEGs). 1,184 genes were upregulated in the G1 subgroup, while 1,095 genes were upregulated in the G2 subgroup (Figure 3A and Supplementary Table S3). GSEA enrichment analysis based on hallmark and KEGG pathway annotations revealed that inflammatory response, interferon-alpha response, interferon-gamma response, and tyrosine metabolism pathways were enriched in the G1 subgroup, and the G2 subgroup was related to MYC targets, KRAS signaling, E2F targets, and G2M checkpoint (Figures 3B,C and Supplementary Table S4). To determine the potential compounds that target the relevant biological functions and pathways, CMap mode of action (MoA) analysis was used to generate candidates based on DEGs. 56 kinds of molecules were able to elevate or repress the expression levels of the DEGs, which was surmised and displayed in Supplementary Table S5 and Supplementary Figure S1B. 21 compounds including 17-beta-estradiol, alpha-estradiol, coumestrol and so on shared the MoA of Estrogen receptor agonist, and five compounds (CL-82198, doxycycline, PD-166793, UK-356618, and WAY-170523) shared the MoA of metalloproteinase inhibitor. All above results indicated the unsupervised hierarchical clustering generated distinct different subgroups based on 19 RA metabolism-related enzymes.

Both G1 and G2 subgroups contain only three genes with more than 10% mutation rate in sarcomas. The top 15 most frequently mutated genes were illustrated in Supplementary Figure S2A and Supplementary Figure S2B. Interestingly, TP53, ATRX, RB1, and MUC16 occupy the top four positions in both cohorts. The mutated positions and frequency of TP53 were similar and showed no survival difference (Supplementary Figure S2C and Supplementary Figure S3). We then calculated the differentially mutated genes using Fisher’s exact test and found that only MAGEC1 showed a significantly different mutation rate between G1 and G2 subgroups. Furthermore, we investigated the exclusive mutations and co-occurring of the top 20 most frequently mutated genes and found some unique co-occurring cases between these two subgroups, such as CTNND2-ARFGEF1 in G1 subgroups and LRP1B-CSMD1 in G2 subgroups.

The tumor microenvironment contains stromal cells, tumor cells, and immune cells. The higher the stromal score and immune score, the higher immune infiltration and stroma infiltration of the tumor. As shown in Figures 3D–F, G1 presented a higher stromal score, immune score, and ESTIMATEScore. On the contrary, G2 had a lower stromal score, immune score, and ESTIMATEScore, which is consistent with the results of pathway analysis between G1 and G2 subgroups. Since bone and soft tissue sarcomas can arise from a variety of body sites, TIMER is the only method that takes tissue specificity into account when estimating immune cell populations (Li et al., 2020), we used TIMER to further characterize the immune cell infiltration in sarcomas (Supplementary Figure S4). In distinct RA-based subtypes, we found that there were more CD4^+^ and CD8^+^ T cell infiltration in G1 subgroups than in G2 (Figure 3G). Besides, we have shown in GSEA enrichment analysis that inflammatory response was enriched in G1 subgroups, suggesting that tumor microenvironment with G1 RA expression characteristics not only infiltrate with a greater amount of anti-tumor immunocytes but also these immune cells are immunoreactive and explain why patients in the G1 group have a better prognosis.

### Role of RA Signature Genes in Predicting Prognosis for Sarcoma

**FIGURE 4:**
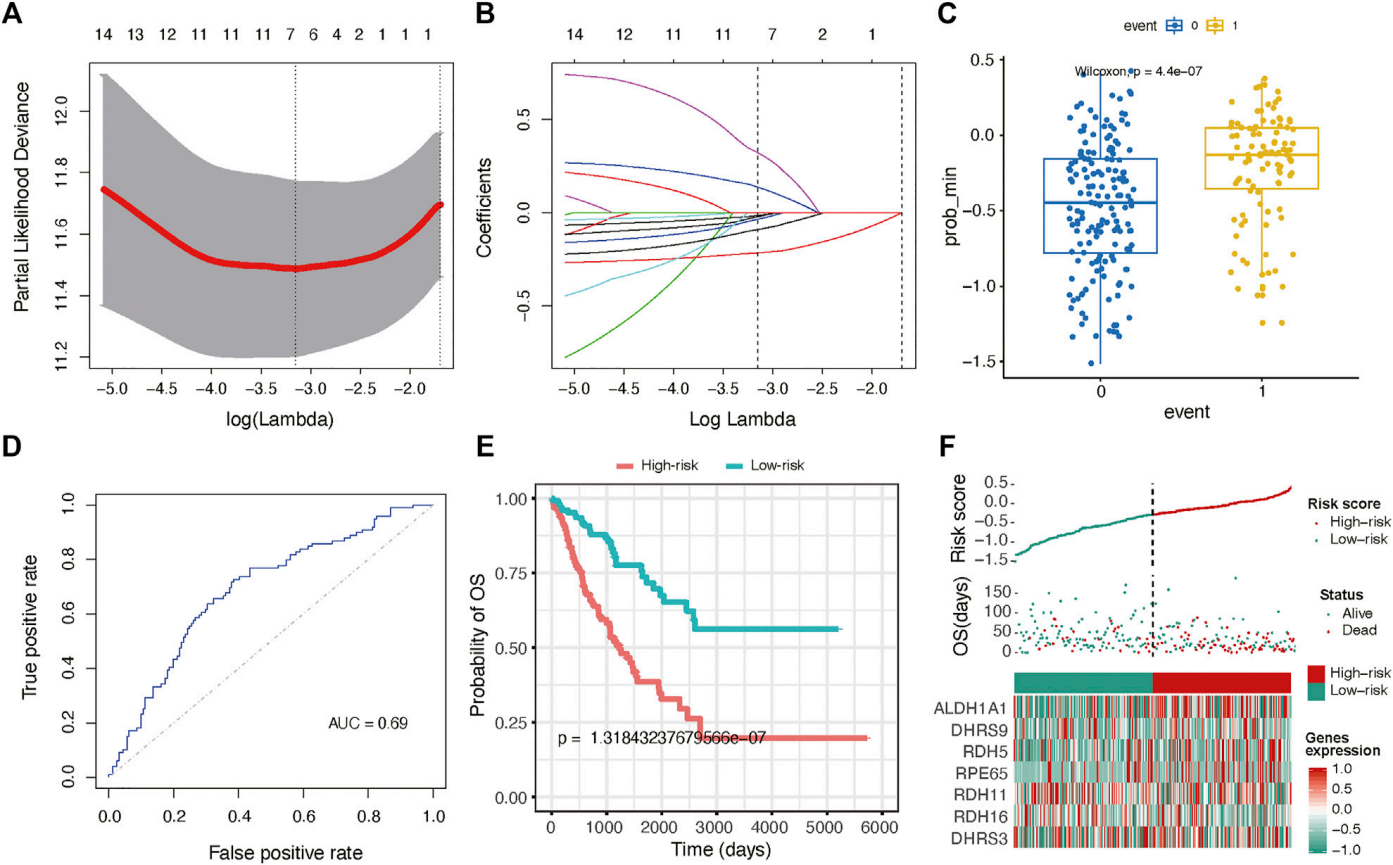
Gene signature with RA metabolism-related enzymes. The regression coefficients. **(A)** calculated by multivariate Cox regression using LASSO **(B)** are exhibited **(C)** Boxplot shows the risk score difference between samples with different OS statuses. **(D)** ROC curves show the predictive efficiency of the risk signature. **(E)** Kaplan–Meier survival curves for patients in the TCGA dataset assigned to high- and low-risk groups based on the risk score. **(F)** Risk plot for the SARC patients in TCGA datasets. Each panel consists of three rows: the top row showed the risk score distribution for the high- and low-risk score group; the middle row represents the SARC patients’ distribution and survival status; the bottom row shows that the heatmap of seven prognostic metabolism-related enzymes expression.

To expanse the application of RA metabolism-based enzymes in different kinds of datasets, we generated a prognostic model to score all samples. The 19 putative RA metabolism-related enzymes were exploited to construct the model. LASSO Cox regression algorithm was adopted. After 1,000-time alteration and cross-validation, a 7-gene (ALDH1A1, DHRS9, RDH5, RPE65, RDH11, RDH16, DHRS3) based risk signature was finally used. The parameters of the model were displayed in Figure 4A and Figure 4B. Significant differences were observed between different OS status groups Figure 4C. To evaluate the predictive performance of the model, we calculated AUC and the result was shown in Figure 4D. The AUC was 0.69 in predicting overall survival status and was able to reach 0.75 in survival time-based ROC analysis (Figure 4D and Supplementary Figure S5). Cox proportional hazard regression analysis indicated the risk signature was an independent prognostic factor (Figure 4E). A similar trend was observed in three independent bone and soft tissue validation datasets (TARGET, GSE17679 and GSE21050) with significant different survival risk (*p* = 0.0005 for TARGET dataset, *p* = 0.075 for GSE17679 dataset and *p* = 0.032 for GSE21050 dataset) (Supplementary Figure S6). In addition, the risk score was found to be significantly related to unsupervised hierarchical clustering results of TCGA samples with an AUC of 0.903, showing a robust predictive ability of RA metabolism-related enzymes (Supplementary Figure S7).

**FIGURE 5:**
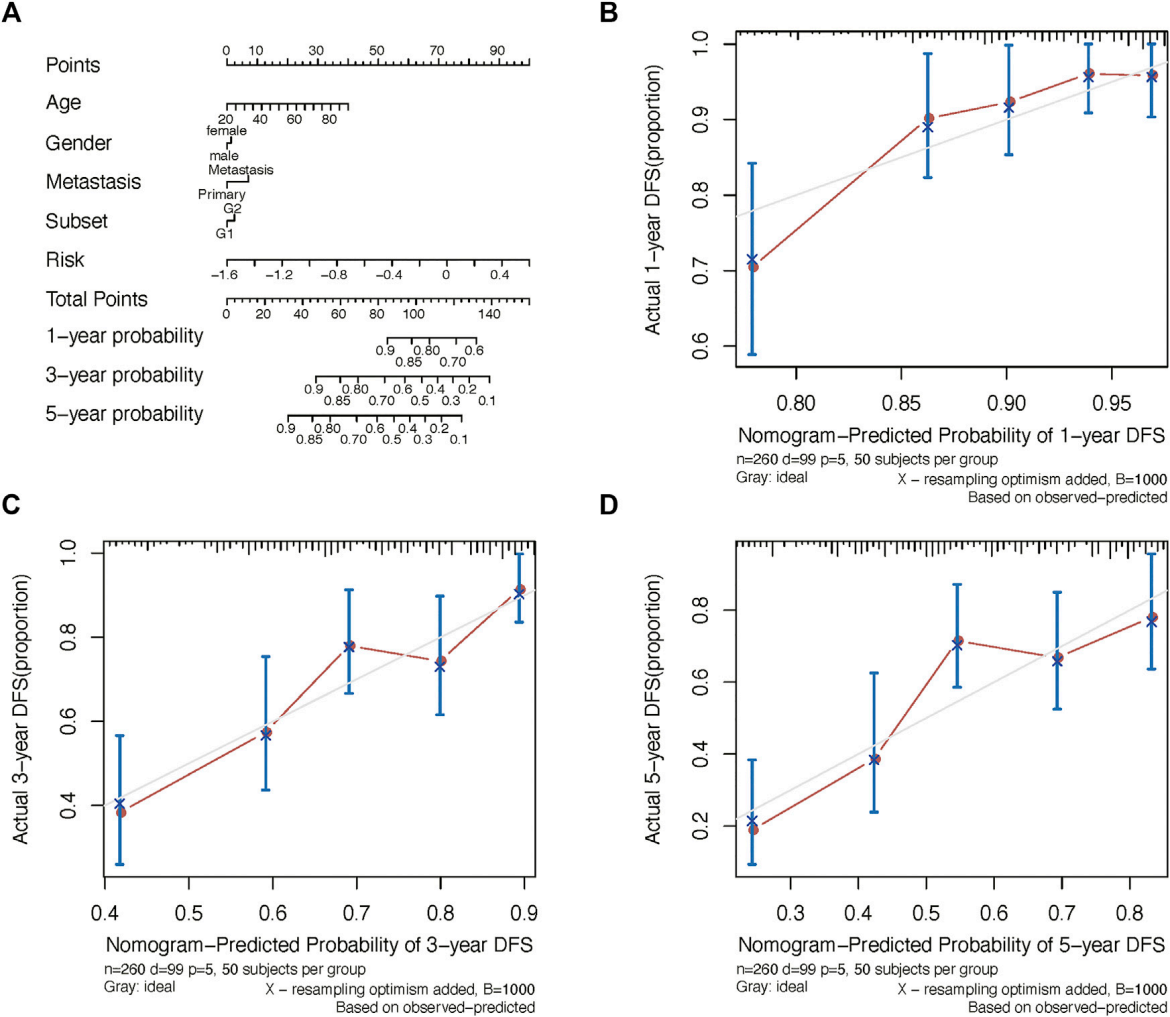
Nomogram analysis. **(A)** Nomogram composed of age, gender, and risk score for the prediction of 1-, 3-, and 5-years OS probability. Calibration plot for the evaluation of the nomogram in predicting 1-year **(B)**, 3-years **(C)**, and 5-years **(D)** OS probability.

We further constructed a nomogram to predict 1-, 3-, and 5-years OS probability based on the risk score. As shown in Figure 5A, Nomogram analysis indicated risk score deviated very little from actual OS probability, such as 1-, 3- and 5-years OS probability (Figures 5B–D).

**FIGURE 6:**
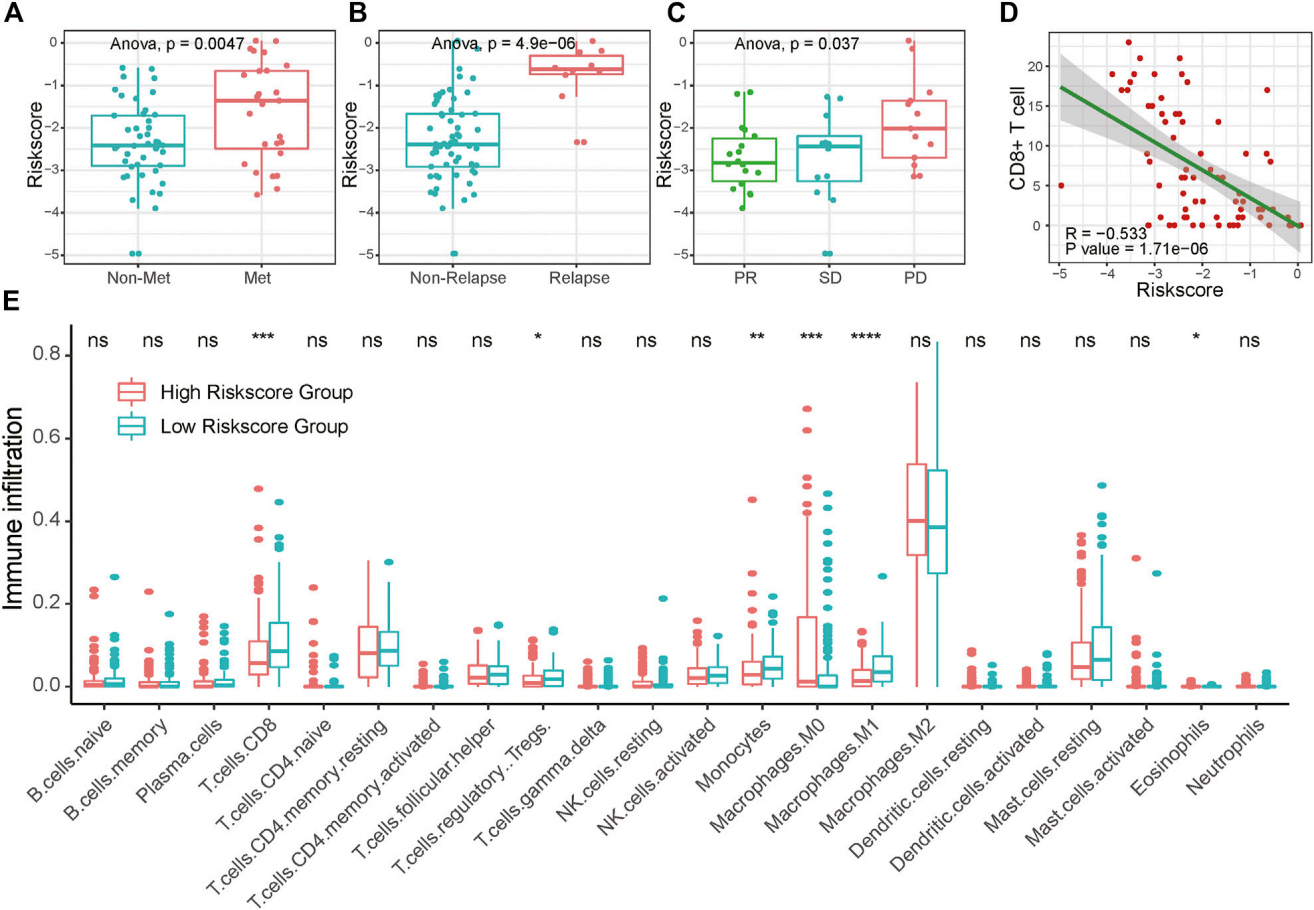
Association of RA risk score and sarcoma prognostic indicators. **(A–B)** Lung metastasis and local recurrence sarcoma patients had higher risk scores than those without tumor dissemination or untreated tumor. **(C)** Sarcoma patients with unfavorable neoadjuvant chemotherapy responses link to the higher risk score **(D)** CD8^+^ T cell infiltration negatively correlated with the RA risk score **(E)** The fractions of immune cells between high-risk score and low-risk score samples in TCGA database by using CIBERSORT. PD, progressive disease; PR, partial response; SD, stable disease.

Presenting with lung metastasis or local recurrence indicates worse overall survival for sarcoma patients. For further clinical verification, we calculated the risk score for 71 bone and soft tissue sarcoma patients base on these seven signature genes expression in biopsy tissue samples using RT-qPCR. The clinicopathological characteristics of 71 sarcoma patients in this research are listed below in Supplementary Table S7. We found that patients with distant metastasis or recurrence tumors had higher risk scores than those without tumor dissemination or untreated tumor (Figures 6A,B). Moreover, patients with unfavorable neoadjuvant chemotherapy responses linked to higher risk scores (Figure 6C; Supplementary Figure S8). Similar trends were also found in bone sarcoma patients and soft tissue sarcoma patients separately (Supplementary Figure S11). Together, these results suggested that the potential application of the risk score as a valuable marker to improve the estimation of sarcomas’ prognosis.

### High RA Based Risk Score Correlated With Suppressive Sarcoma Immune Microenvironment

To investigate whether the RA-based risk score is related to the immune microenvironment of sarcoma, we characterized cell composition using CIBERSORT (Supplementary Table S6). 18 types of immune cells were decomposed and used to calculate the correlation coefficient with RA based risk score. As shown in Figure 6E and Supplementary Figure S9, the fractions of CD8^+^ T cells, Treg cells, Monocytes, and Macrophages were negatively correlated with a risk score. To avoid algorithm bias, we compared the results with the other two immune cell fraction calculation tools ImmuneCellAI and xCell. Different tools showed a similar phenomenon that the fraction of CD8^+^ T cells were negatively correlated with risk scores. What’s more, increased CD8^+^ T cells fraction seemed to be related to central memory T cell and effector memory T cell. A higher amount of naïve CD8^+^ T cells was found in the high-risk score group compared to the low-risk score group, suggesting that increasing RA-based risk score was associated with the immunosuppressive tumor microenvironment (Supplementary Figure S10). We further validated the connection between RA-based risk score and the immune tumor microenvironment in 71 clinical samples of sarcoma using CD8 immunohistochemical staining. Consistently, RA based risk score was negatively correlated with the number of tumor CD8^+^ T cell infiltration (Figure 6D and Supplementary Figure S8).

## Discussion

Sarcomas are a broad family of mesenchymal malignancies exhibiting histologic diversity. The classification of sarcoma we currently use in making clinical decisions and stratifying patients mainly indicates tumor cells origin. Nevertheless, as a result of the high levels of heterogeneity discovered in sarcoma (Hatina et al., 2019), the prognosis between patients may vary significantly despite similar origins (Merry et al., 2021). Recent studies demonstrated that the dysregulation of retinoic acid signaling in the sarcoma mouse model reprograming the tumor immunologic microenvironment and thus leading to an unfavorable response to treatment and poor prognosis (Balkwill, 2020; Devalaraja et al., 2020). However, a comprehensive analysis of retinoic acid metabolism abnormality in realistic sarcoma patients is still lacking, which restricted the use of retinoic acid-related therapy strategy in the clinic.

By systematically analyzing the molecular features of 19 retinoic acid metabolism-related enzymes in 263 patients’ samples from the TCGA database, we found that the majority of RA metabolism-related enzymes were differentially expressed between sarcomas and normal tissues. Using an unsupervised hierarchical clustering method, we stratified sarcoma patients and subdivided them into two distinct subtypes with different metabolic statuses and prognoses. Gene set enrichment analysis indicated that several immune pathways were enriched in the subtype G1 subgroup, suggesting that higher infiltration of immune cells which was associated with prolonged survival (Chew et al., 2012; Gajewski et al., 2013). Whereas, the G2 subgroup was related to oncogenic pathways such as MYC targets and KRAS signaling. The MYC oncogene is a global regulator of the immune response (Casey et al., 2018). KRAS abnormal tumor cells can switch the tumor immune microenvironment from an antitumorigenic to a pro-tumorigenic (Dias Carvalho et al., 2018). The immunosuppressive cancer microenvironments in the G2 subgroup allow sarcoma cells to escape from immunosurveillance and continue growing and thus leads to tumor recurrence, metastasis, and or failure of chemotherapy or checkpoint inhibitors of immunotherapy. These results suggest that tumor RA metabolism is closely related to patients’ prognosis and immunity.

Practical tools to understanding the role of RA metabolism in sarcoma patients are valuable to improve the estimation of sarcomas prognosis and therapy decision making. To this end, we developed a seven RA metabolism-related enzymes-based model for sarcoma patients to predict prognosis. Validation using two independent sarcoma datasets have shown its considerable predictive ability. Moreover, in 71 bone and soft tissue sarcoma primary tumor samples collected in our hospital, we found that high-risk patients tend to develop distant metastasis, had higher local recurrence rates and unfavorable neoadjuvant chemotherapy responses, all of these factors indicate patients’ unsatisfied prognosis. Therefore, high RA risk sarcoma patients may need more frequent monitoring. Meanwhile, we observed that low-risk patients’ tumor samples presented more amount of cytotoxic antitumor CD8^+^ T cells infiltration, suggesting that such patients may benefit from immunotherapy.

To our knowledge, the present study is the first comprehensive retinoic acid metabolism base molecular classification for sarcoma patients. The feasible and powerful metabolic index risk model may facilitate selection for appropriate neo-chemotherapy designs, tailoring of follow-up protocols, and therapy decision-making for different subgroups of patients with sarcoma.

## Materials and Methods

### Data acquisition

TCGA and TARGET RNA sequence level 3 normalized data and clinical information of SARC were downloaded from UCSC Xena (https://xenabrowser.net/datapages/) for further analysis. The RNA-seq data of GSE17679 and GSE21050 datasets were downloaded from Gene Expression Omnibus (GEO) database (https://www.ncbi.nlm.nih.gov/gds/). The RNA-seq data of healthy human tissue was downloaded from the Genotype-Tissue Expression (GTEx) database (http://commonfund.nih.gov/GTEx/).

### Selection of RA Metabolism-Related Enzymes

The RA metabolism-related enzymes data set was collected in three major aspects. The major and important method was searching the literature from PubMed. And besides, the enzyme information from the MetaCyc database (Caspi et al., 2020) and KEGG retinol metabolism pathway were integrated.

### Bioinformatics Analysis

The protein interaction network was constructed based on GeneMANIA (https://genemania.org/). Unsupervised hierarchical clustering of SARC samples was achieved by R package “ConsensusClusterPlus v1.42.0”. PCA was constructed to reveal the expression pattern difference of SARC. The R package “survival v3.1-17” (https://cran.r-project.org/web/packages/survival/) was adopted to acquire the overall survival through Kaplan-Meier estimation. We calculated the fold change and adjusted *p-*value by the DESeq2 v1.18.1 package for all genes between different groups, in which an adjusted *p-*value less than 0.05 and |log2FoldChange| > 1 was considered the differential expression gene. Gene Set Enrichment Analysis (GSEA) was used to classify the functional pathway difference between different subgroups. mode of action (MoA) analysis was generated based on the analysis of differentially expressed genes in Connectivity Map (Lamb et al., 2006) (https://clue.io/). Compounds with an absolute value of *p* < 0.05 and enrichment ≥0.7 were treated as potential therapeutic drugs for different subgroups in SARC. The mutation data of SARC was downloaded from Xena Browser (https://xenabrowser.net/datapages/) and the R package “maftools v2.2.20” was used to dissect the mutation pattern differences. CIBERSORT (Chen et al., 2018), ImmuneCellAI, and xCell were used to retrieve the immune cell components in SARC samples.

### Gene Signature Identification

Univariate Cox regression analysis of the expression of 19 RA metabolism-related enzymes was performed on SARC samples to determine the candidate genes with R package “glmnet v2.0-18”. Finally, a total of 7-gene signatures was obtained after 1,000-time alteration and cross-validation. The risk score for each patient was calculated with the linear combinational of the signature gene expression. The regression coefficients were determined by the minimum criteria. That is, Risk Score = k1*x1+k2*x2+…+ki*xi (i = n). where i represents each selected enzyme, k is the regression coefficient and x is the expression level. In this paper, the final model was defined as Risk Score = -0.007*RPE63–0.020*DHRS3 - 0.007*DHRS9 - 0.210*ALDH1A1 + 0.113*RDH11 + 0.280*RDH16 - 0.078*RDH5. We further classified the samples into a high-risk group and a low-risk group based on the median value of risk scores.

### Patients and Tissue Samples

Primary tumor samples were collected from 71 bone and soft tissue sarcoma patients at Sun Yat-sen University Cancer Center, from Nov 2017 to Oct 2019. Definite diagnosis was confirmed histopathologically by two experienced pathologists. Clinicopathological characteristics of 71 bone and soft tissue sarcoma patients in this research are listed below in Supplementary Table S7. RECIST1.1 criteria were used to estimate the curative effect of neoadjuvant chemotherapy mainly based on the change of tumor volume according to MRI of the primary site and the development of metastatic lesions through the chest CT. This research was approved by the Institutional Ethics Committee at Sun Yat-sen University Cancer Center (GZR 2019-283).

### Reverse Transcription-PCR and Real-Time RT-PCR

Total RNA from osteosarcoma cell lines and tissue samples was purified using the TRIZOL (Invitrogen, Carlsbad, CA, United States) according to the product instructions, and the first-strand cDNA was synthesized by First Strand cDNA Synthesis Kit (Fermentas, Glen Burnie, MD), following the manufacturer’s protocol. Real-time RT-PCR was carried out using SYBR^®^ qPCR Mix (Toyobo). Primers (5’to3′) used in this study are listed below.

**Table:** 

ALDH1A1-F	CGG​GAA​AAG​CAA​TCT​GAA​GAG​GG
ALDH1A1-R	GAT​GCG​GCT​ATA​CAA​CAC​TGG​C
DHRS9-F	CCA​GAG​AAT​GTC​AAG​AGG​ACT​GC
DHRS9-R	CTG​TAG​TCC​TCT​AGT​GTC​AGC​C
RDH5-F	CTG​TGA​CCA​ACC​TGG​AGA​GTC​T
RDH5-R	GAT​GCG​CTG​TTG​CAT​TTT​CAG​GT
RPE65-F	TTT​GGC​ACC​TGT​GCT​TTC​CCA​G
RPE65-R	GTT​GGT​CTC​TGT​GCA​AGC​GTA​G
RDH11-F	AGC​AGG​TGT​TGG​TGC​GGA​AAC​T
RDH11-R	CGG​ACA​CAT​CAT​CAC​TCC​TGC​A
RDH16-F	TAT​GGC​GTG​GAA​GCC​TTC​TCT​G
RDH16-R	GGT​CCC​AAA​TCT​CCA​GGA​AGC​T
DHRS3-F	GGG​CAC​TGA​GTG​CCA​TTA​CTT​C
DHRS3-R	CGG​CAT​TGT​TCA​CCA​GGA​TGG​T

After quantified the expression of seven enzymes used for model construction, we calculated the risk score for each sample based on the linear model mentioned above.

### Immunohistochemistry, and CD8^+^ T Cell Infiltration

Tissue slides from paraffin-embedded tissue were dewaxed using xylene, hydrated in different gradient ethanol, and then subjected to citrate buffer-induced antigenic epitope retrieval. Goat serum buffer (ZSGB-BIO, Beijing) was applied to the sections on the slides for non-specific antigenic blocking. The sections were then incubated with primary antibody CD8 (1:100, ZSGB-BIO, #ZA-0508) overnight at 4 °C. After incubating with secondary antibodies (ZSGB-BIO, Beijing), DAB substrate solution was applied to reveal the color of antibody staining, and hematoxylin was counterstained on the slides by immersing sides.

## Data Availability

Publicly available datasets were analyzed in this study. This data can be found here: Publicly available datasets were analyzed in this study. These data can be found at: The Cancer Genome Atlas (TCGA), Datasets link: https://xenabrowser.net/datapages/. And Gene Expression Omnibus (GEO), Datasets link: https://www.ncbi.nlm.nih.gov/gds/. And the Genotype-Tissue Expression (GTEx), Datasets link: http://commonfund.nih.gov/GTEx/. The RDD number for this dataset is RDDB2021319935.

## Abbreviations

ADH, alcohol dehydrogenases; DEGs, differentially expressed genes; GEO, Gene Expression Omnibus; GSEA, Gene Set Enrichment Analysis; GTEx, Genotype-Tissue Expression; IFN-γ, interferon-gamma; MoA, mode of action; RA, Retinoic acid; RALDH, retinal dehydrogenases; RARRES2, Retinoic-acid receptor responder protein 1; RARβ, retinoic acid receptor beta; TGF-β, transforming growth factor-beta.
