# Supplementary figures and images for: Retinoic Acid Metabolism-Related Enzyme Signature Identified Prognostic and Immune Characteristics in Sarcoma
Source: Front Cell Dev Biol. 2022 Feb 3;9:780951. doi: 10.3389/fcell.2021.780951 (PMC8852678; doi:10.3389/fcell.2021.780951)

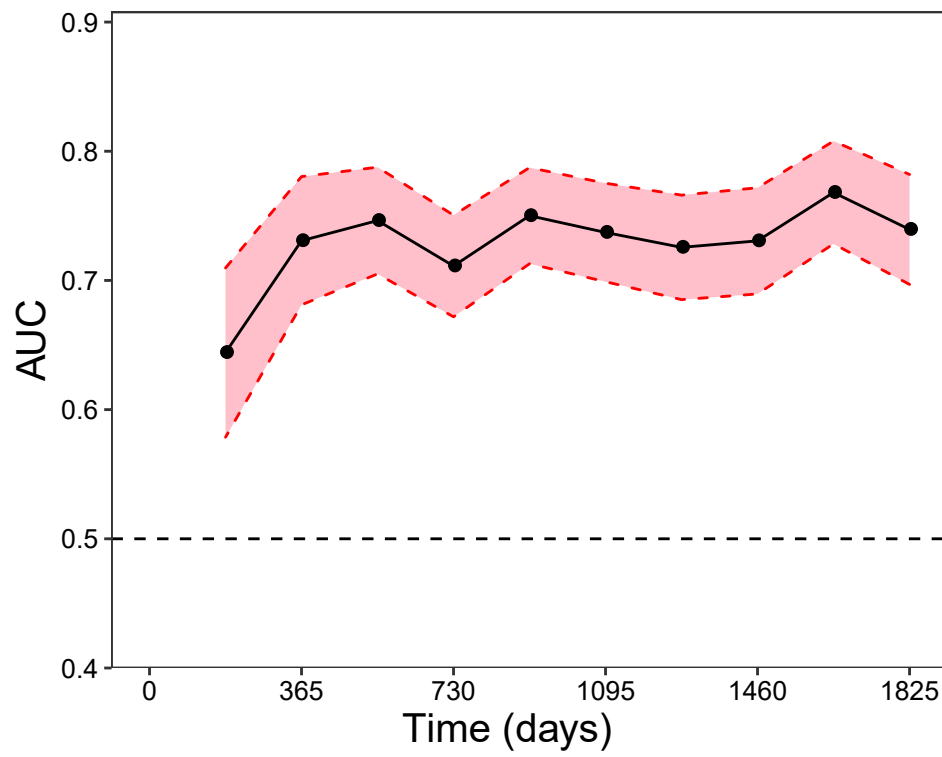

Supplement: Supplementary file 3 [file Image5.pdf]

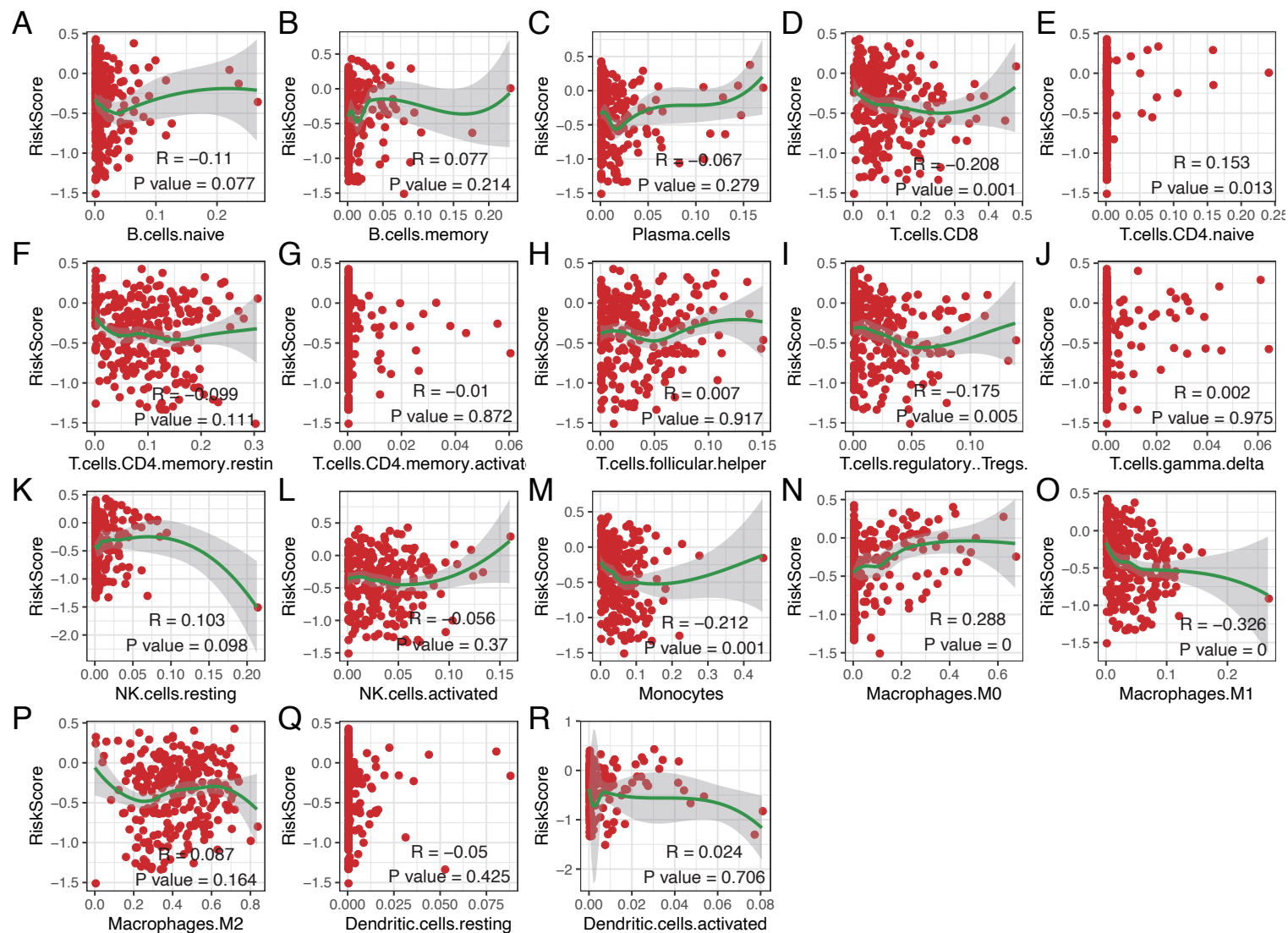

Supplement: Supplementary file 4 [file Image9.pdf]

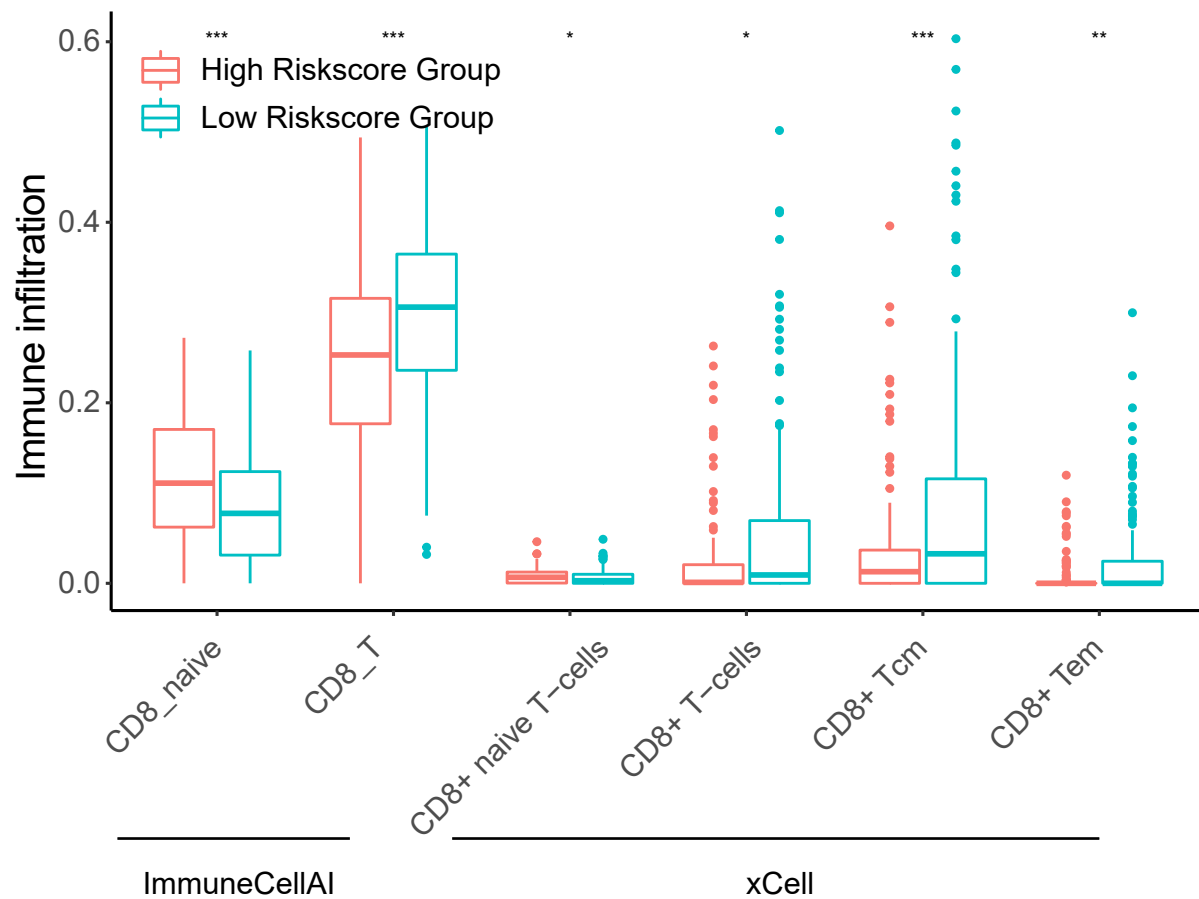

Supplement: Supplementary file 6 [file Image10.pdf]

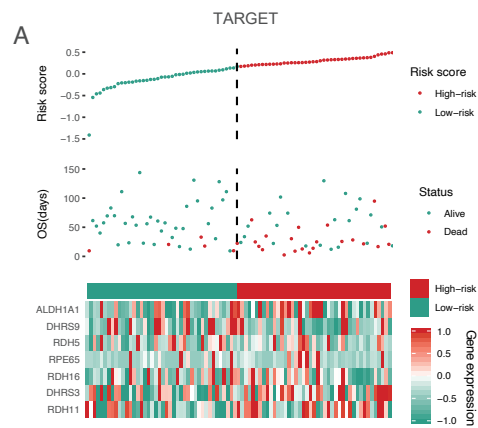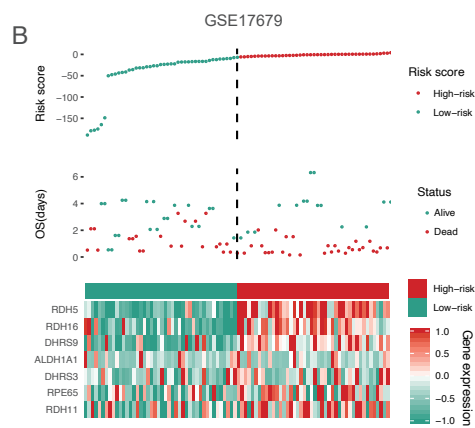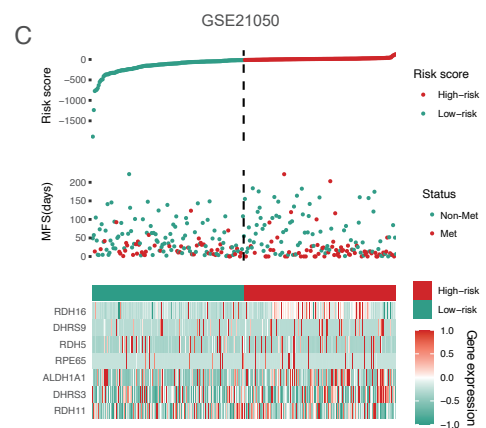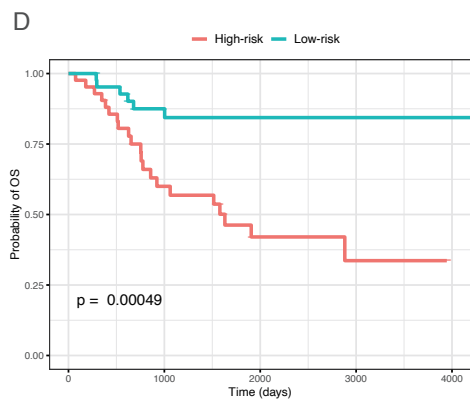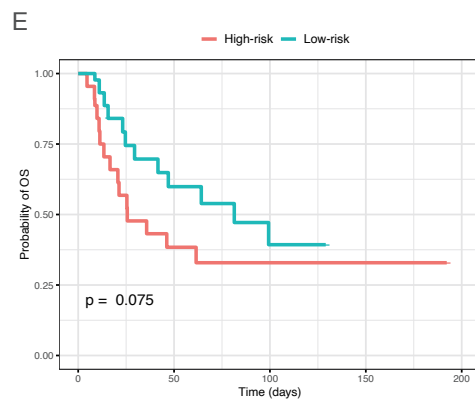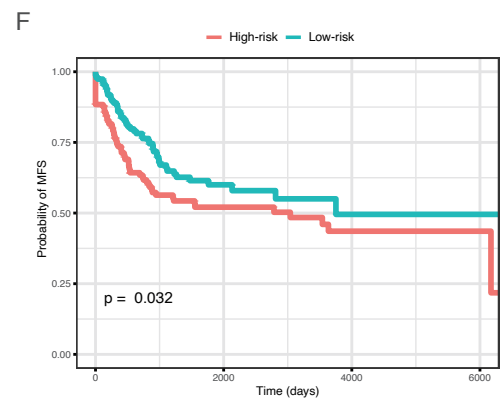

Supplement: Supplementary file 7 [file Image6.pdf]

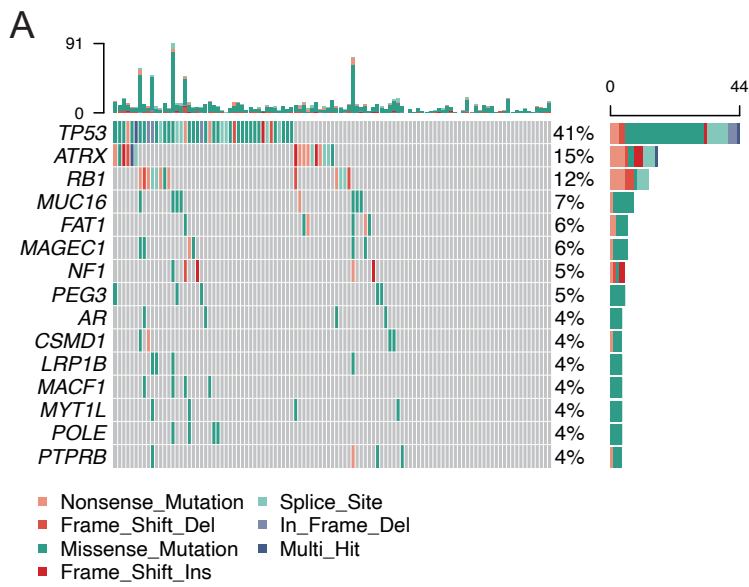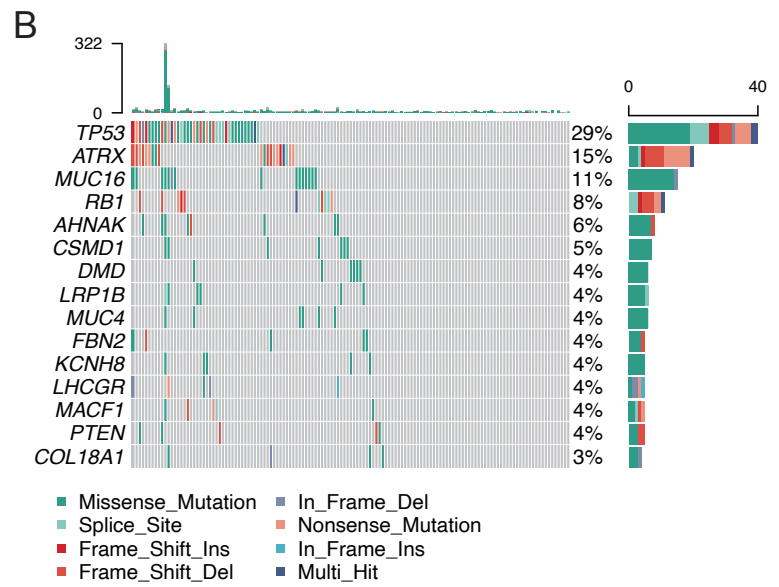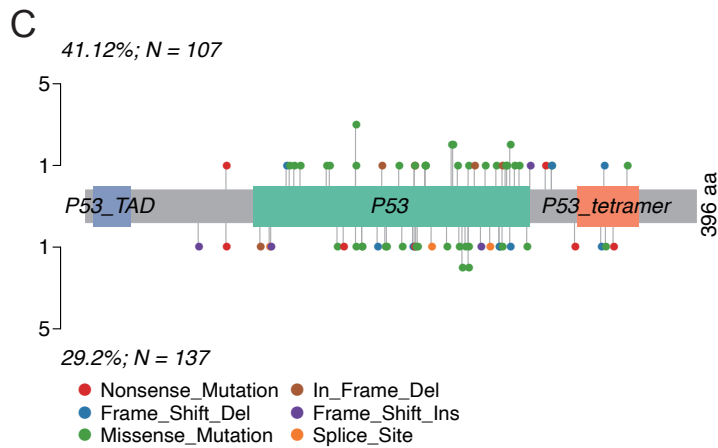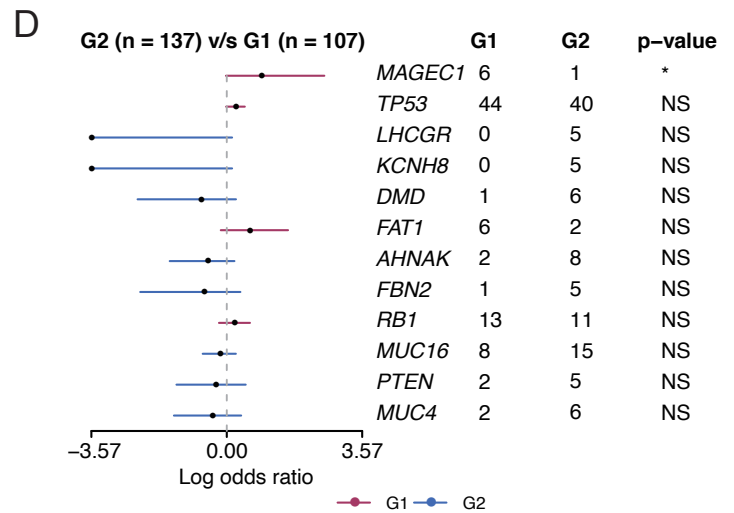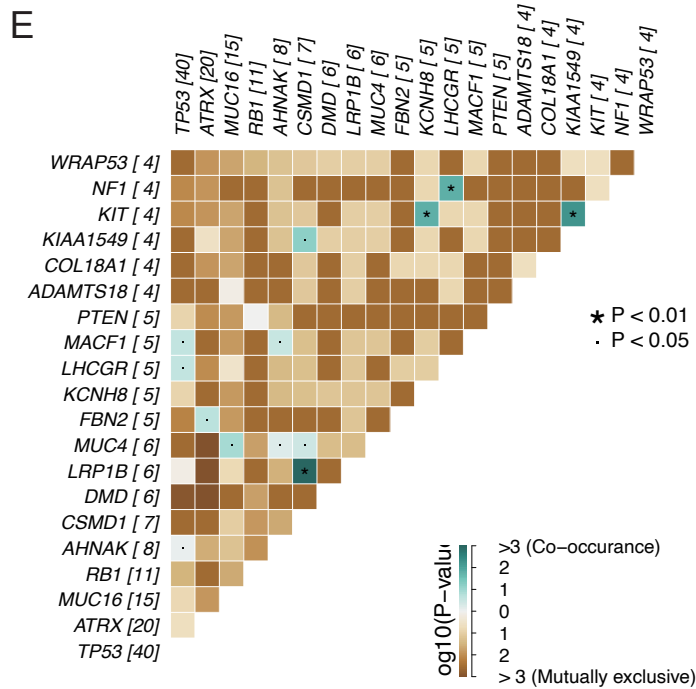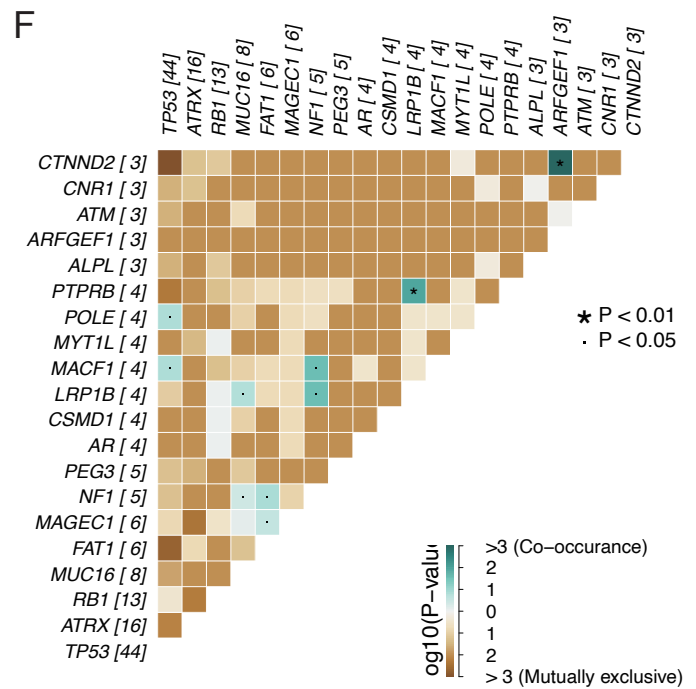

Supplement: Supplementary file 9 [file Image2.pdf]

A

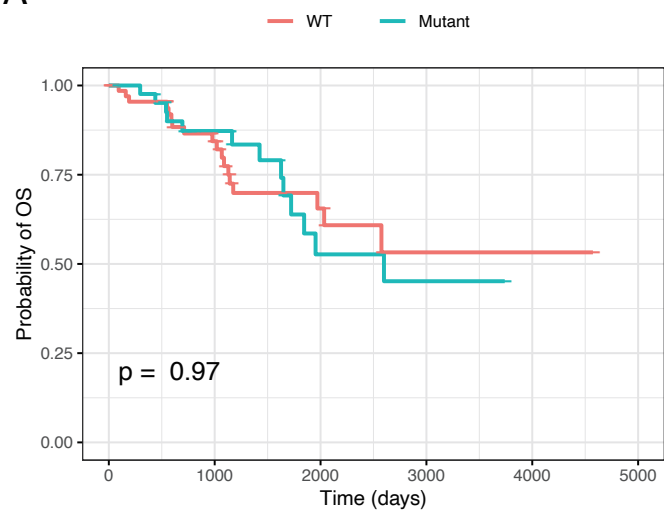

B

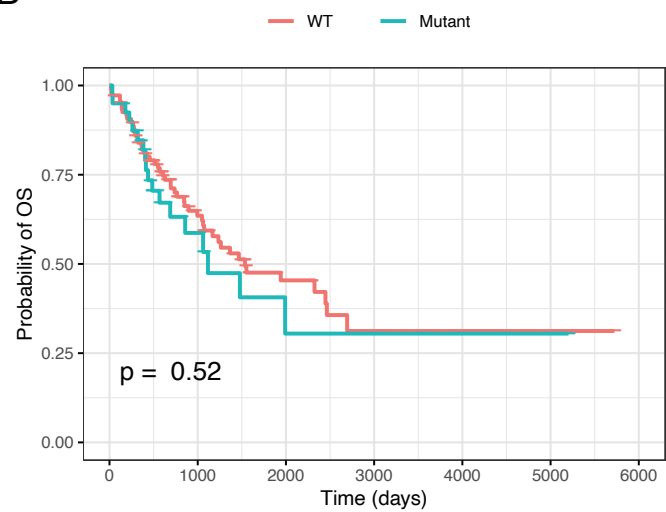

Supplement: Supplementary file 11 [file Image3.pdf]

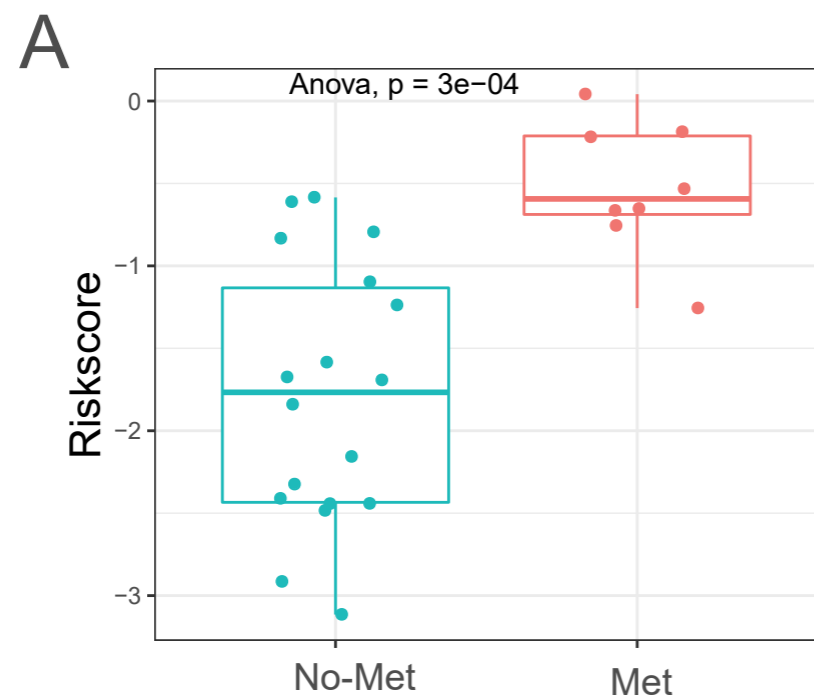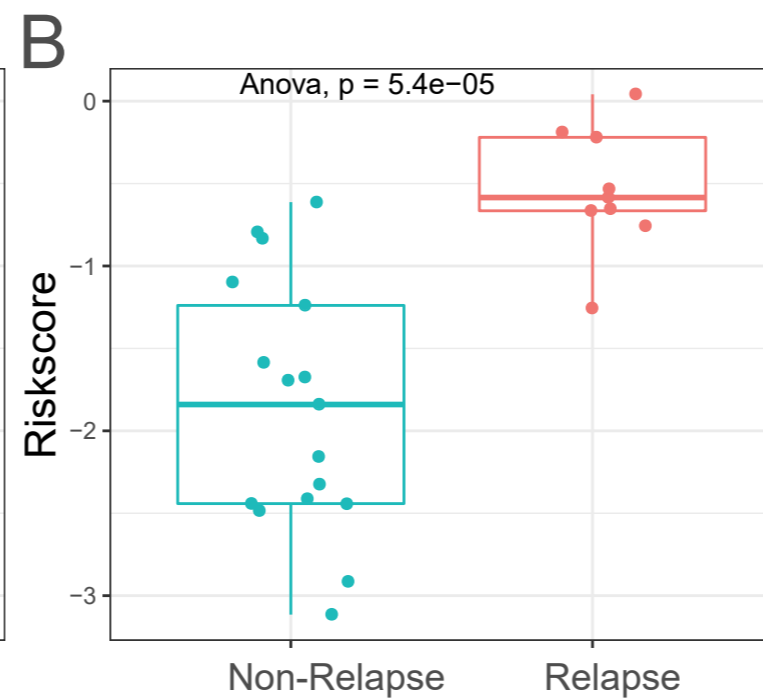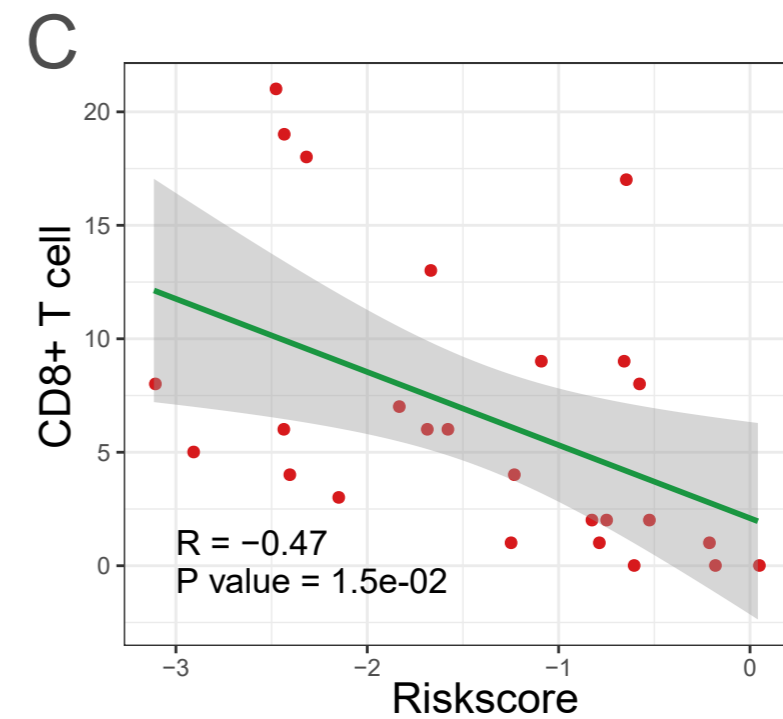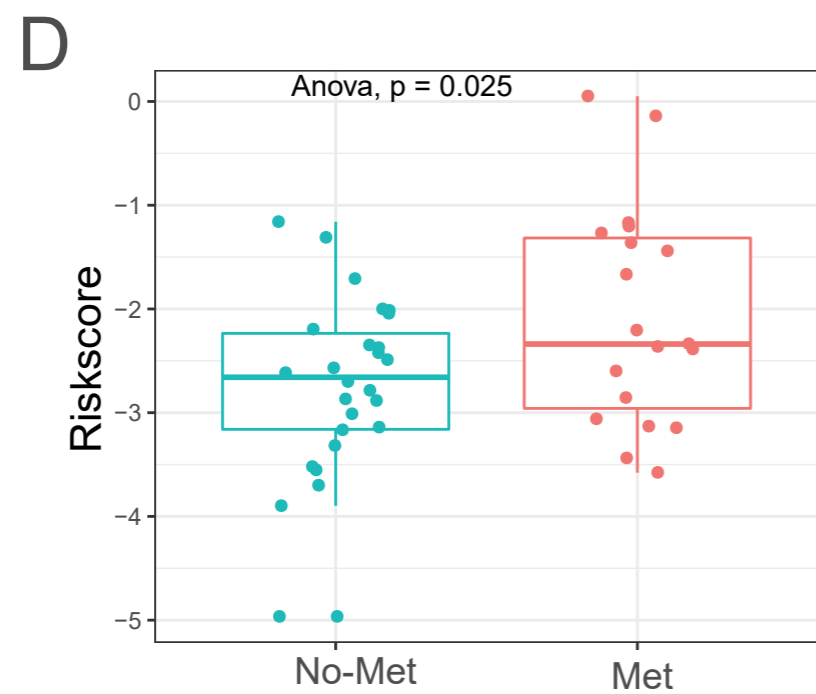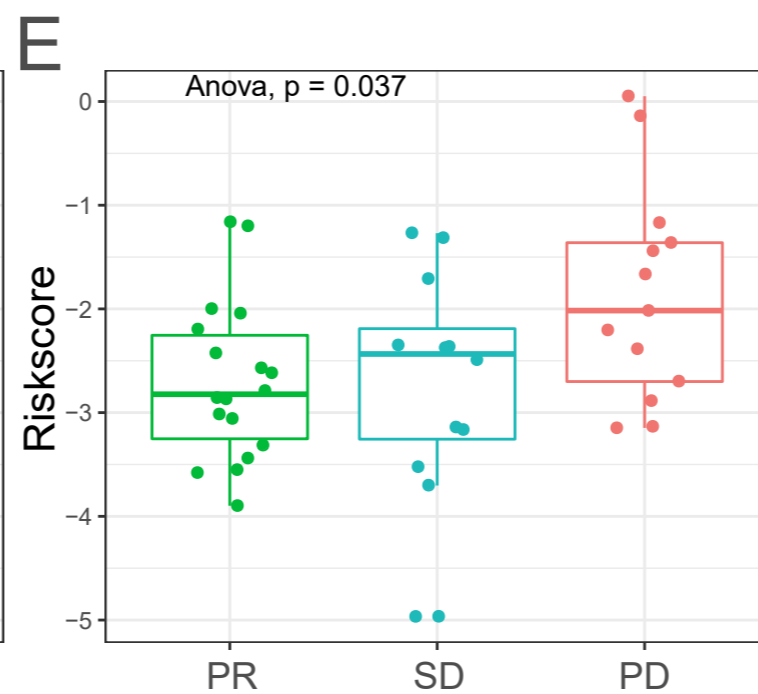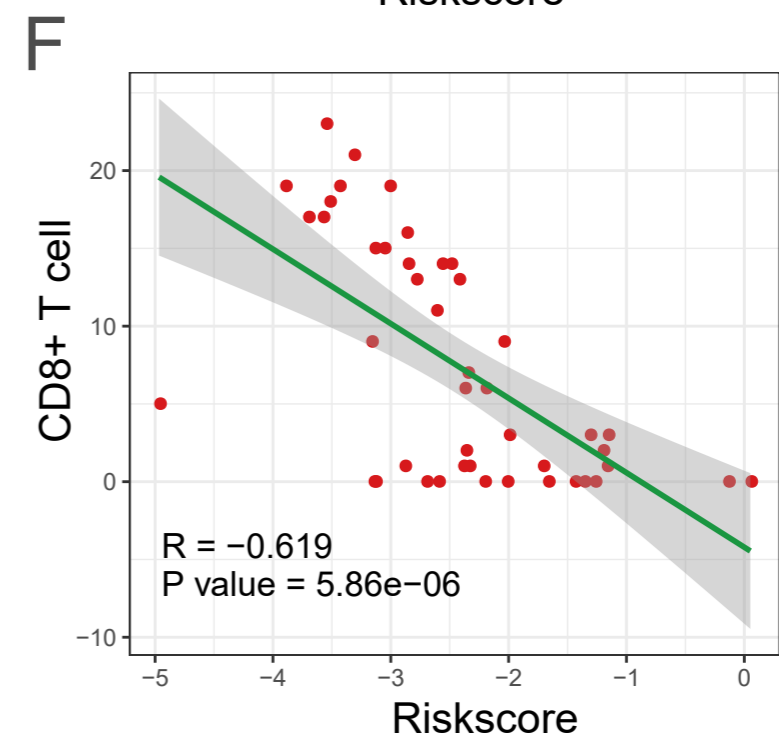

Supplement: Supplementary file 14 [file Image11.pdf]

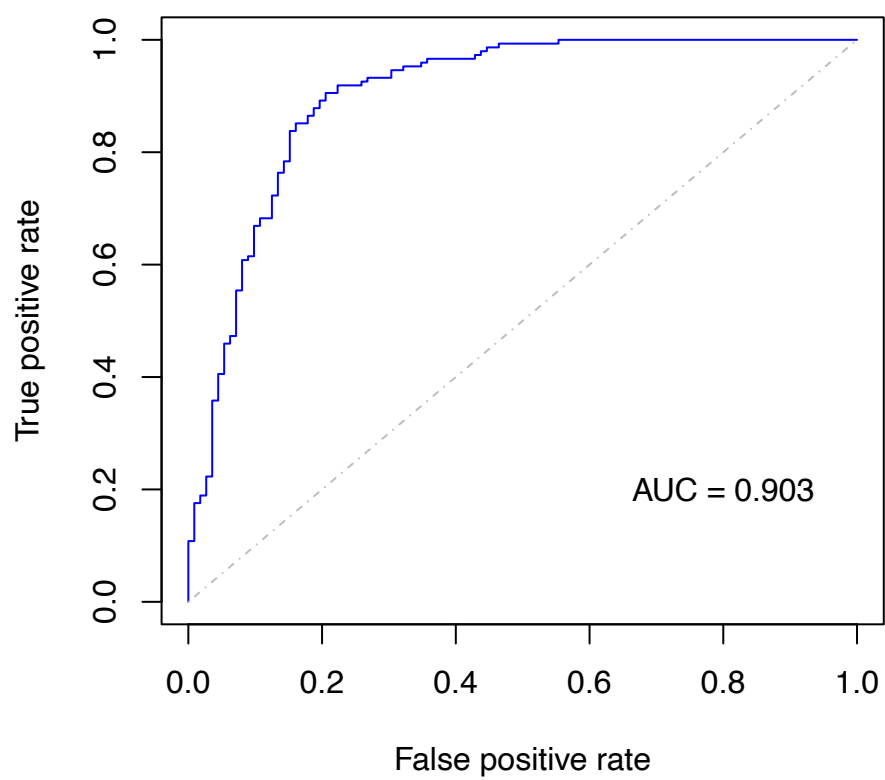

Supplement: Supplementary file 15 [file Image7.pdf]

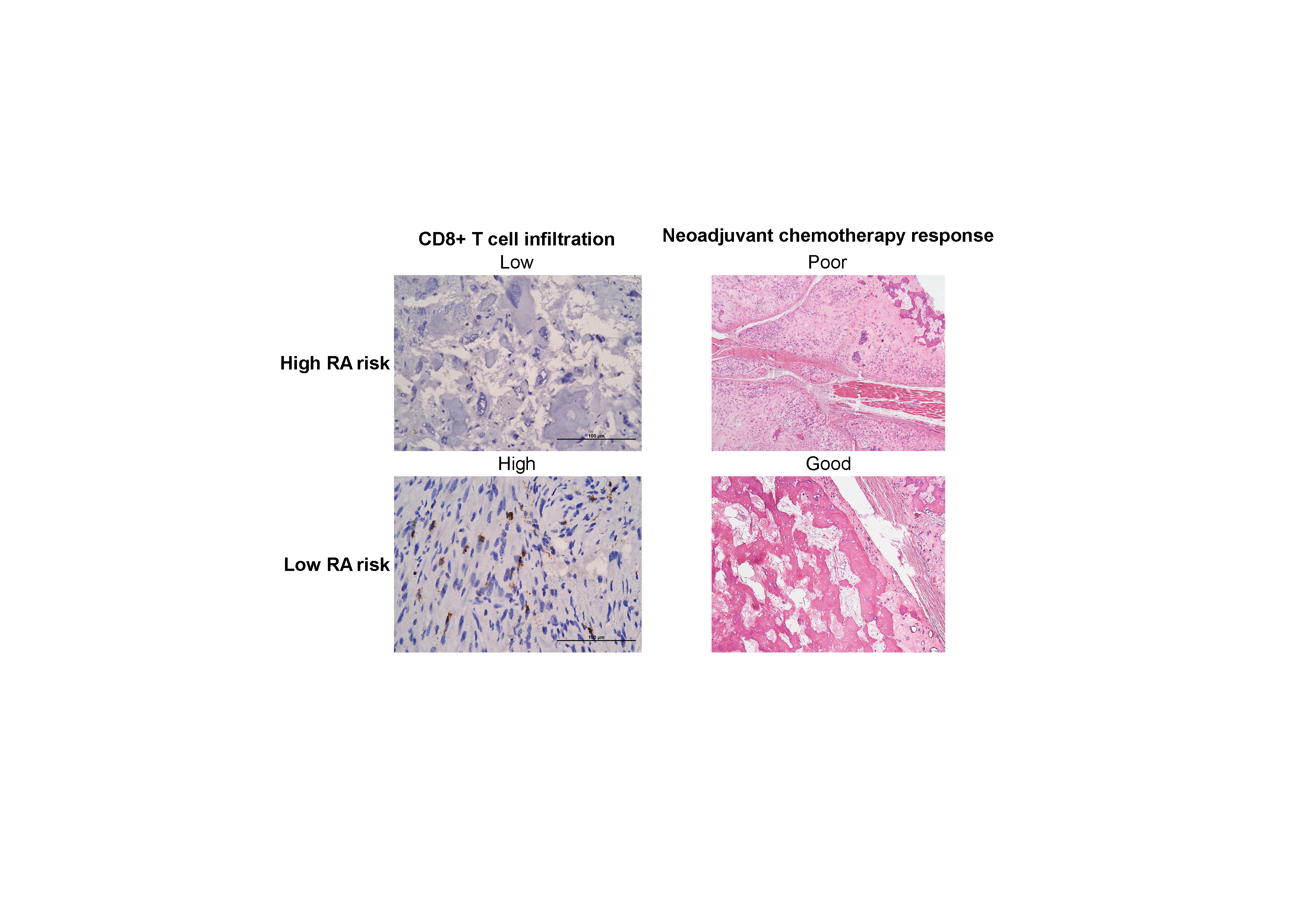

Supplement: Supplementary file 16 [file Image8.tif]

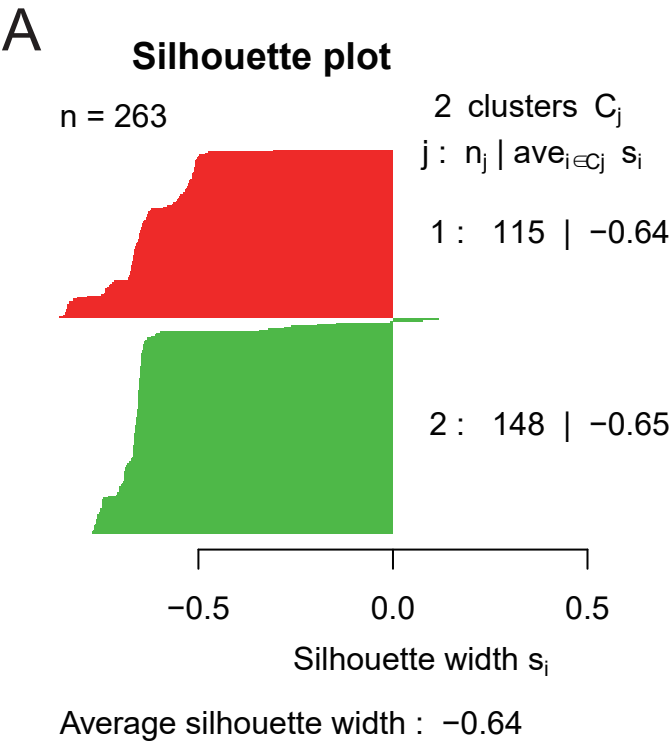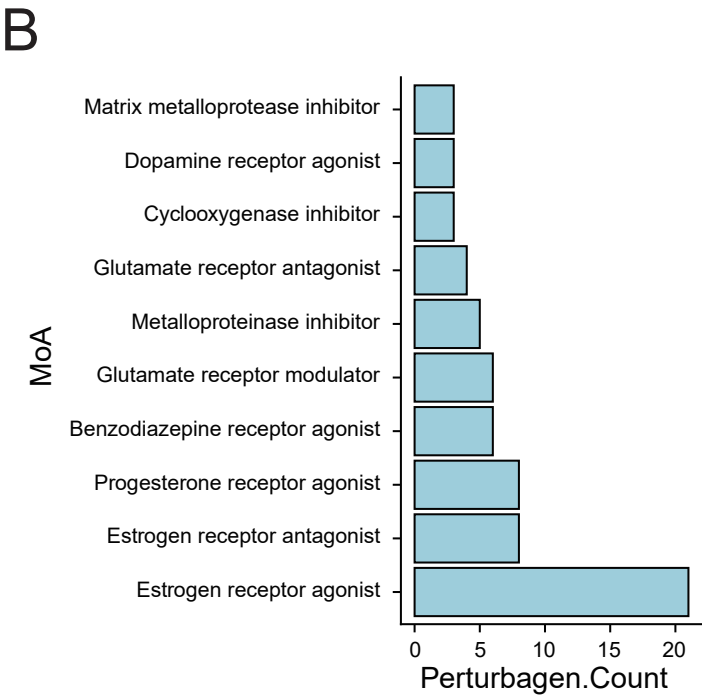

Supplement: Supplementary file 19 [file Image1.pdf]
