# Supplementary material for: Retinoic Acid Metabolism-Related Enzyme Signature Identified Prognostic and Immune Characteristics in Sarcoma
Source: Front Cell Dev Biol. 2022 Feb 3;9:780951. doi: 10.3389/fcell.2021.780951 (PMC8852678; doi:10.3389/fcell.2021.780951)
Supplement: Supplementary file 5 [file Table8.docx]

**Supporting Information**

**Supplemental Figures**

**Figure S1.** (A) Silhouette widths of the subtypes. (B) The number of compounds in the top 10 MoA.

**Figure S2.** **Landscape of Somatic Mutation in G1 and G2 Cohorts.** (A and B) Waterfall plot shows the mutation distribution of the top 15 most frequently mutated genes. (C) The lollipop plot illustrates the differential distribution of variants for TP53. (D) Forest plot displays the top 12 most significantly differentially mutated genes between two cohorts. (E and F) The heatmap illustrates the mutually co-occurring and exclusive mutations of the top 25 frequently mutated genes.

**Figure S3.** Kaplan-Meier curves show the independent relevance between overall survival time and TP53 mutation in G1 and G2 subgroups. (A and B)

**Figure S4.** Immune cell score heat map, where different colors represent the expression trend in different samples in TCGA-SARC dataset.

**Figure S5.** ROC curves show the time-dependent predictive efficiency of the risk signature.

**Figure S6.** Risk plot for the SARC patients in TARGET, GSE1679 and GSE21050 datasets.

**Figure S7.** ROC curves show the predictive efficiency of the risk signature on TCGA sarcoma samples.

**Figure S8.** HE staining shows the neoadjuvant chemotherapy responses in patients with different risk scores after chemotherapy. IHC shows the infiltration of cytotoxic CD8 cells of samples with different risk scores. Scale bars: 100 μm.

**Figure S9.** Correlation of the immune cell members and risk score.

**Figure S10.** Correlation of the CD8+ T cell infiltration and risk score based on ImmuneCellAI and xCell.

**Figure S11.** **Association of retinoic acid risk score and sarcoma prognostic indicators separately.** (A-C) Association of retinoic acid risk score and bone sarcoma prognostic indicators. (D-F) Association of retinoic acid risk score and soft tissue sarcoma prognostic indicators.

**Supplemental Tables**

**Table S1.** Evaluation of metrics performance among different numbers of subgroups.

**Table S2.** The distribution of clinical variables for G1 and G2 subgroups.

**Table S3.** Differentially expressed genes between G1 and G2 groups.

**Table S4.** GSEA enrichment results based on KEGG and hallmark gene sets.

**Table S5.** Connective Map screened 56 potential small molecules specific to different sub-groups.

**Table S6.** Immune cell components derived from CIBERSORT.

**Table S7.** Clinicopathological characteristics of 71 sarcoma patients.
