# Supplementary material for: Retinoic Acid Metabolism-Related Enzyme Signature Identified Prognostic and Immune Characteristics in Sarcoma
Source: Front Cell Dev Biol. 2022 Feb 3;9:780951. doi: 10.3389/fcell.2021.780951 (PMC8852678; doi:10.3389/fcell.2021.780951)
Supplement: Supplementary file 8 [file Image4.pdf]

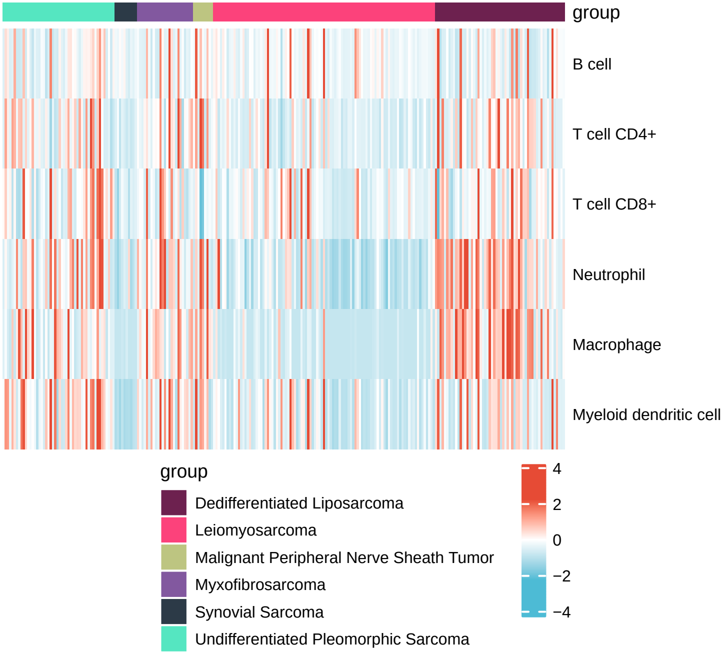
group
B cell
T cell CD4+
T cell CD8+
Neutrophil
Macrophage
Myeloid dendritic cell
group
Dedifferentiated Liposarcoma
Leiomyosarcoma
Malignant Peripheral Nerve Sheath Tumor
Myxofibrosarcoma
Synovial Sarcoma
Undifferentiated Pleomorphic Sarcoma
4
2
0
−2
−4
